# New-onset ulcerative colitis temporally associated with with ixekizumab in a psoriasis patient successfully treated with guselkunmab: A case report

**DOI:** 10.1097/MD.0000000000046924

**Published:** 2026-01-02

**Authors:** Jing Tang, Wenjuan Li, Haiyan Zhang

**Affiliations:** a The Second Clinical Medical College of Guangzhou University of traditional Chinese Medicine, Guangdong, China; b Guangdong Hospital of Traditional Chinese Medicine, Guangzhou, China.

**Keywords:** case report, guselkumab, psoriasis, ulcerative colitis

## Abstract

**Rationale:**

Ulcerative colitis (UC) and psoriasis are both chronic inflammatory diseases mediated by the immune system. Ixekizumab, used to treat psoriasis, carries a risk of inducing or exacerbating UC. Infliximab has also been reported to exacerbate psoriasis. Guselkumab (GUS) can be used to treat both UC and psoriasis. Currently, there are few case reports on the use of GUS for the treatment of UC–psoriasis comorbidity. Our observations support the rationale for IL-23–targeted agents in patients with overlapping psoriasis and inflammatory bowel disease, and they provide exploratory evidence that the psoriasis-labeled regimen of GUS may suffice for selected UC patients.

**Patient concerns:**

A 51-year-old woman with a history of psoriasis for more than 20 years was diagnosed with ulcerative colitis due to abdominal pain, diarrhea, and mucous-bloody stools while using ixekizumab. During treatment with infliximab for ulcerative colitis, her psoriatic lesions worsened. Switching to guselkumab achieved complete skin clearance (PASI 0) and sustained endoscopic remission of UC (Mayo 1, UCEIS 1) without the need for additional biologic therapy.

**Diagnoses:**

Psoriasis has been previously diagnosed; ulcerative colitis was diagnosed based on the patient’s clinical symptoms, colonoscopy, and pathological examination.

**Interventions:**

The patient was given guselkumab (100 mg every 8 weeks) and mesalazine (4 g every day).

**Outcomes:**

Four months after starting guselkumab, the patient’s psoriasis area and severity index score reached 0, and guselkumab therapy was continued thereafter. Since then, she has experienced no gastrointestinal symptoms, and her psoriasis has been well-controlled. On June 28, 2025, a follow-up colonoscopy was performed. The Mayo endoscopic score was 1, and the ulcerative colitis endoscopic index of severity score was 1.

**Lessons:**

Dermatologists closely monitor gastrointestinal symptoms in patients with psoriasis who are receiving ixekizumab to ensure safe medication use. Patients with ulcerative colitis and psoriasis may actively consider using guselkumab.

## 1. Introduction

Ulcerative colitis (UC) and psoriasis are both chronic inflammatory diseases mediated by the immune system.^[1]^ Several common gene loci and overlapping inflammatory pathways have been reported between psoriasis and inflammatory bowel disease (IBD). Tumor necrosis factor-alpha (TNF-α) inhibitors, such as infliximab (IFX), have been used to treat UC; however, TNF-α inhibitors can induce or exacerbate psoriasis.^[2]^ Ixekizumab is a biologic drug approved for the treatment of psoriasis and psoriatic arthritis. By specifically binding to interleukin-17A, ixekizumab inhibits its activity, thereby modulating the immune response and alleviating the clinical manifestations of psoriasis. Nevertheless, evidence suggests that ixekizumab is strongly associated with the onset of IBD.^[3–6]^ Guselkumab (GUS), a fully human IgG1λ monoclonal antibody, is a selective inhibitor of the IL-23p19 subunit. It was the first drug in its class to be approved for the treatment of adult patients with moderate to severe plaque psoriasis. Since 2024, it has been approved for adult patients with moderate-to-severe active UC. Herein, we report a case of new-onset UC temporally associated with ixekizumab in a patient with psoriasis, successfully treated with GUS.

## 2. Case presentation

**Figure 1.**
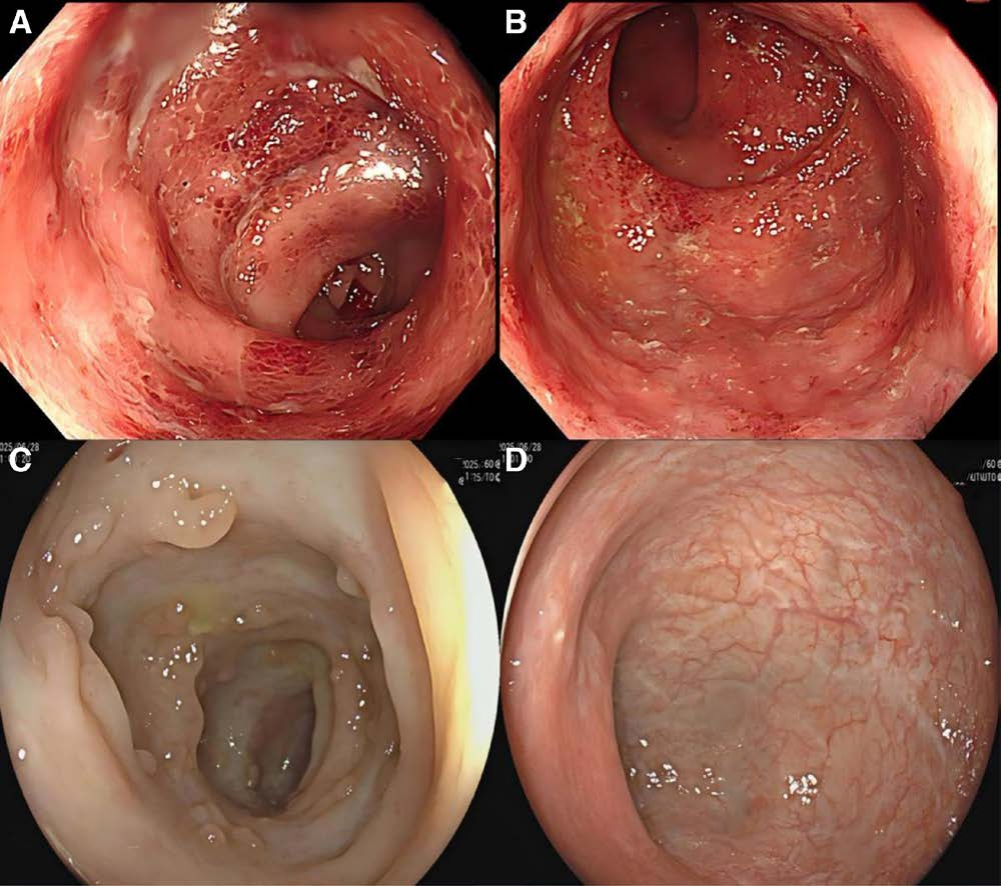
(A and B) Colonoscopy performed at our hospital in May 2023 showed lesions in the rectum and sigmoid colon. (C and D) Colonoscopy in June 2025 revealed white scar-like changes and polypoid hyperplasia in the mucosa, with no ulcers or erosion observed.

A 51-year-old woman with a history of psoriasis for more than 20 years had been regularly using ixekizumab to treat her condition since August 2022, achieving reasonable disease control. During ixekizumab therapy, the patient developed abdominal pain and diarrhea in February 2023, followed by mucous-bloody stools beginning in April 2023. In May 2023, the patient was admitted to our department for inpatient treatment. Laboratory results indicated: C-reactive protein (CRP): 30.87 mg/L, erythrocyte sedimentation rate (ESR): 50 mm/hour (Table 1). Colonoscopy findings revealed that loss of the mucosal vascular pattern from the sigmoid colon (approximately 18 cm from the anal verge) to the anal. The mucosa was friable with contact bleeding, had a granular appearance, and showed patchy erosions with a small amount of purulent exudate. The lesions were continuous (Fig. 1A and B) Histopathological examination revealed focal erosion of the sigmoid colon and rectal mucosa, a reduced number of crypts, and slight disorganization of their arrangement. In addition, cryptitis and crypt abscesses were observed. After completing additional tests and examinations to exclude other types of colitis, a diagnosis of UC (E2, S2) was made. She was initially treated with mesalazine (4 grams every day) for 2 months, but her intestinal symptoms did not improve satisfactorily. After evaluation, treatment was changed to IFX. After excluding contraindications, IFX (300 mg intravenously) was administered on July 7, July 21, and August 18, 2023, corresponding to cycles 1 to 3, respectively. This resulted in an improvement in the patient’s condition. The patient’s CRP decreased to 0.51 mg/dL, and the ESR decreased to 16 mm/hour (Table 1). During the IFX treatment period, ixekizumab was discontinued. In September 2023, the patient experienced a recurrence of psoriatic lesions, characterized by scattered erythema and scales visible throughout the body, particularly around the waist, accompanied by itching. Following assessment by a dermatologist, psoriasis treatment was switched to GUS at 100 mg every 8 weeks. Four months after starting GUS, the patient’s psoriasis area and severity index score reached 0, and GUS therapy was continued thereafter. During GUS treatment, the patient discontinued IFX and switched to mesalazine (4 grams every day) for UC. Since then, she has experienced no gastrointestinal symptoms, and her psoriasis has been well-controlled. On June 28, 2025, the patient underwent a follow-up examination, with CRP at 5.74 mg/L and ESR at 24 mm/hour (Table 1). The colonoscopy results showed the mucosa of the sigmoid colon, approximately 25 cm from the anus, appeared pale with multiple white scar-like changes and polypoid hyperplasia. No erosion, ulcers, or narrowing were observed, although the vascular patterns were unclear. Approximately 10 to 15 cm from the anus, the mucosa showed white scar-like changes. At 10 cm from the anus, scattered red patches were observed on the local mucosa, with unclear vascular patterns (Fig. 1C and D). No erosion, ulcers, or polypoid hyperplasia were present. The Mayo endoscopic score was 1, and the UC endoscopic index of severity score was 1. The patient continues to receive treatment with GUS (100 mg every 8 weeks) combined with mesalazine (4 grams/day). During this treatment period, the patient has demonstrated excellent compliance, regularly returning to the hospital for follow-up appointments and medication refills, and has not experienced any drug-related adverse events (Table 2).

**Table 1:** Laboratory test results.

	CRP mg/L	ESR mm/h	WBC 10^9^/L
May 18, 2023	30.87	50	10.08
July 7, 2023	17.96	46	6.11
July 21, 2023	0.34	24	5.95
August 18, 2023	0.51	16	5.47
May 22, 2023	5.74	24	6.71

CRP = C-reactive protein, ESR = erythrocyte sedimentation rate, WBC = white blood cells.

**Table 2:** Treatment timeline.

Event	Time
Intestinal symptoms flare up	The patient developed abdominal pain and diarrhea in February 2023, followed by mucous-bloody stools beginning in April 2023
Improvement in gastrointestinal symptoms	August 18, 2023
Psoriasis flares up	September 2023
Psoriasis remission	January 2024
First colonoscopy	May 2023
Colonoscopy during follow-up	June 28, 2025
Duration of ixekizumab use	August 2022 to May 2023
IFX usage time	July 7, 2023 to August 18, 2023
GUS start date	September 12, 2023
Montreal classification	E2, S2

GUS = guselkumab, IFX = infliximab.

## 3. Discussion

UC and psoriasis are both chronic inflammatory diseases mediated by the immune system.^[1]^ Several common gene loci and overlapping inflammatory pathways have been reported between psoriasis and IBD, including IL-23, IL-17, tumor necrosis factor, and T-helper 17 cells. The present case involves a patient with a history of psoriasis for over 20 years who developed UC during treatment with ixekizumab. Ixekizumab is a humanized immunoglobulin G4 monoclonal antibody that selectively binds to IL-17, preventing its interaction with its receptor. Since its approval in 2017 for the treatment of psoriasis, psoriatic arthritis, and ankylosing spondylitis, multiple cases of IBD have been reported,^[3–6]^ most of which occurred during maintenance treatment with ixekizumab. Considering that ixekizumab may induce or exacerbate IBD, it is recommended that dermatologists closely monitor gastrointestinal symptoms in patients with psoriasis who are receiving ixekizumab to ensure safe medication use.

UC is a chronic, nonspecific inflammatory disease of the intestine, and TNF-α inhibitors are among the main drugs used for its treatment. However, for psoriasis, while TNF-α inhibitors can be used in treatment, they may also induce new-onset psoriasis or exacerbate existing stable psoriasis. This phenomenon known as “paradoxical psoriasis,” with IFX being the most commonly reported agent.^[2,7,8]^ Although the mechanism of action tied to the development of psoriatic lesions has yet to be fully elucidated, there are several well-supported theories on the mechanism by which TNF-α inhibitors result in psoriatic lesion development. The most accepted theory is that the TNF-α inhibitor leads to uncontrolled production of IFN-α by pDCs. As a result, excess levels of IFN-α activate myeloid dendritic cells, which then stimulate pathogenic T cells. This results in the release of the inflammatory cytokines IL-23, TNF-α, and IL-12, which activate T-helper cells, stimulate further inflammatory cytokine release and result in unregulated keratinocytes. Inhibition of TNF-α can result in unregulated IFN-α production by pDCs and, in turn, the development of psoriatic lesions.^[9,10]^ This may explain why the patient’s psoriasis worsened after 3 courses of IFX treatment. However, during the course of IFX treatment, the patient discontinued the biologic agent controlling psoriasis disease following assessment by a dermatologist, switching to topical medications and oral Chinese herbal decoctions for maintenance therapy. Given the patient’s history of poor response to previous oral medications, discontinuation of ixekizumab may also lead to recurrence and worsening of psoriasis disease.

GUS is a fully human IgG1λ monoclonal antibody that selectively inhibits the IL-23p19 subunit. It is approved for treating moderate to severe plaque psoriasis and active psoriatic arthritis, and has also shown effectiveness in Crohn disease. In May 2025, the China National Medical Products Administration approved GUS for adult patients with moderate-to-severe active UC who have not responded to, or cannot tolerate, conventional therapies or biologics. This marks the first approval of an IL-23 inhibitor for the treatment of UC in China. According to the QUASAR study evaluation, GUS is effective and safe as both induction and maintenance therapy for patients with moderate to severe active UC.^[11,12]^ This patient had previously used oral medications, topical treatments, and red light therapy to manage the psoriasis, but all yielded suboptimal results. After switching to ixekizumab therapy, gastrointestinal symptoms emerged. Following evaluation by a dermatologist, the patient was subsequently switched to GUS. At the initiation of GUS therapy, the patient’s intestinal symptoms were already well controlled. For maintenance treatment of UC and PSA, the GUS dosage was 100 mg q8w. Therefore, this patient did not receive the guideline-recommended starting dose of GUS for UC and psoriasis treatment. Instead, a regimen of GUS q8w combined with mesalazine 4 grams/day was administered. Both the patient’s intestinal and skin symptoms were well controlled, and no adverse reactions occurred. However, clinical decisions should be based on a thorough risk-benefit assessment and carried out with full informed consent.

## 4. Conclusion

Herein, GUS was successfully used to treat UC in a patient with psoriasis. The case highlights that the new-onset UC temporally associated with ixekizumab, while IFX may exacerbate psoriasis symptoms. The GUS UC and psoriasis dosage regimen (100 mg subcutaneously every 8 weeks) successfully achieved remission of both intestinal and skin lesions, suggesting that some UC patients whose clinical symptoms are adequately controlled may not need to initiate treatment with GUS intravenously. Nevertheless, further validation using larger sample sizes is needed. This case may provide a valuable reference for treatment regimens in clinical practice for patients with UC and psoriasis comorbidity.

## Acknowledgments

We are also grateful to all the researchers, including the physicians, nurses, and technicians, who participated in this study.

## Author contributions

**Investigation:** Jing Tang, Wenjuan Li.

**Writing – original draft:** Jing Tang.

**Writing – review & editing:** Haiyan Zhang.
